# The Prodrug Approach: A Successful Tool for Improving Drug Solubility

**DOI:** 10.3390/molecules21010042

**Published:** 2015-12-29

**Authors:** Daniela Hartmann Jornada, Guilherme Felipe dos Santos Fernandes, Diego Eidy Chiba, Thais Regina Ferreira de Melo, Jean Leandro dos Santos, Man Chin Chung

**Affiliations:** Faculdade de Ciências Farmacêuticas, UNESP—University Estadual Paulista, Rodovia Araraquara Jaú Km 01, 14801-902 Araraquara, São Paulo, Brasil; daniela.dhj@gmail.com (D.H.J.); guilhermefelipe@outlook.com (G.F.S.F.); chiba.diego@gmail.com (D.E.C.); trfmelo@gmail.com (T.R.F.M.); santosjl@fcfar.unesp.br (J.L.S.)

**Keywords:** prodrug, solubility, water-soluble prodrugs, solubility of prodrugs, molecular modification

## Abstract

Prodrug design is a widely known molecular modification strategy that aims to optimize the physicochemical and pharmacological properties of drugs to improve their solubility and pharmacokinetic features and decrease their toxicity. A lack of solubility is one of the main obstacles to drug development. This review aims to describe recent advances in the improvement of solubility via the prodrug approach. The main chemical carriers and examples of successful strategies will be discussed, highlighting the advances of this field in the last ten years.

## 1. Introduction

Poor solubility is one of the main problems faced by researchers during drug development. Commonly, even with the use of current computational “filters” to minimize this problem, compounds that are active *in vitro* may lack adequate pharmacokinetic properties and/or may be difficult to formulate [1]. A study conducted with the top 200 oral drug products in Japan, Great Britain, United States and Spain revealed that approximately 37% of drugs had solubilities of less than 0.1 mg/mL. One explanation may include the need for drugs that are highly potent in low doses, however, this issue represents a challenge in drug discovery [2].

Although the prodrug approach is often considered only when the prototype presents unexpected problems, this strategy offers a versatile approach to drug development and should not be considered a last resort. The prodrug approach is a promising molecular modification by which drug developers and designers can modulate drug pharmacokinetics, pharmacodynamics and toxicology [3].

**Figure 1:**
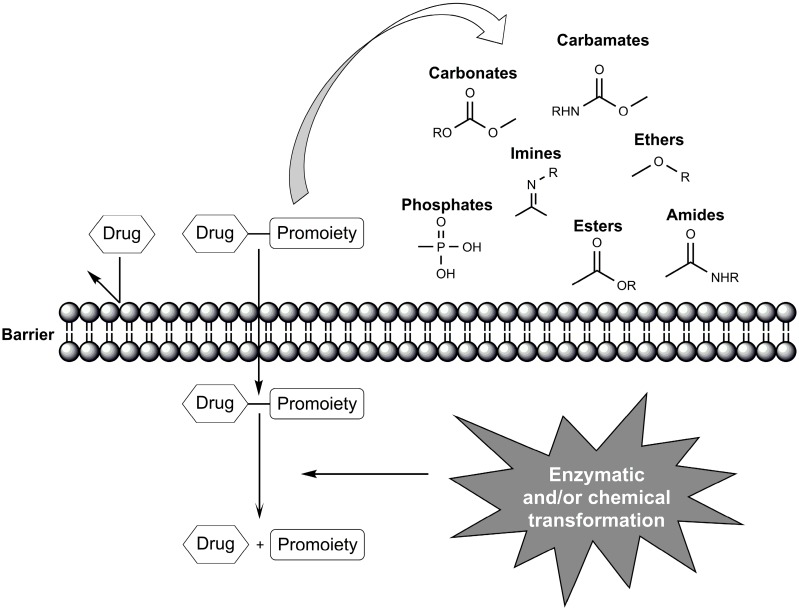
*In vivo* bioactivation of prodrugs by enzymatic and/or chemical transformations.

A prodrug is a poorly active or inactive compound containing the parental drug that undergoes some *in vivo* biotransformation through chemical or enzymatic cleavage, enabling the delivery of the active molecule at efficacious levels [1,3,4]. Prodrugs are conventionally classified in two major classes: carrier-linked prodrugs and bioprecursors. Carrier-linked prodrugs can be classified as bipartite prodrugs, in which the carrier is linked directly to the parent drug, and tripartite prodrugs, in which a spacer links the carrier to the parent drug [5]. Carriers are commonly attached by chemical groups such as ester, amide, carbamate, carbonate, ether, imine, phosphate, among others [1,6,7] (Figure 1). Mutual prodrugs are a type of carrier-linked prodrug in which two active compounds are linked each acting as the carrier to the other. These prodrugs have increased effectiveness through synergistic action [5,8]. Another type of carrier-linked prodrug is the macromolecular prodrug; these prodrugs use polymeric backbones as carriers. Macromolecular prodrugs are commonly used to design prodrugs that will be cleaved inside of a cell and in targeted drug-delivery systems. This approach offers improved drug solubility, stability, drug release and pharmacokinetics. Additionally, it can facilitate the accumulation of a drug at the site of action and improve safety [9,10]. Bioprecursors are inactive compounds that do not have a carrier and are rapidly converted to an active drug after metabolic reactions (normally redox reactions) [5,8].

Undesirable properties, including poor aqueous solubility, chemical instability, insufficient oral or local absorption, fast pre-systemic metabolism, low half-life, toxicity and local irritation are commonly resolved using the prodrug approach. In addition, problems related to drug formulation and delivery can also be overcome by using this strategy [1,11,12,13]. Occasionally, strategies such as particle-size reduction, solubilizing excipients, complexation agents and use of surfactants fail to improve the solubility in water profile and reduce toxicity to desirable levels [13,14]. For example, some surfactants used in parenteral formulations frequently have toxic effects such as anaphylactic reactions [14].

However, in some cases, low-solubility compounds yield false-positive results on *in vitro* assays due to non-specific binding [15]. Moreover, in clinical trials, low-solubility drugs can lead to precipitation and crystalluria, raising additional safety concerns. Poorly soluble drugs have recently been discontinued in clinical assays for this reason [15,16].

For these reasons, the prodrug approach presents a safe and effective strategy by which to improve the solubility of drugs. This review aims to present the latest strategies in prodrug design used to obtain water-soluble compounds for oral and parenteral uses. We selected research from the last ten years (2005 through August of 2015) showing increased solubility through the prodrug approach. In the literature search, we used the following terms: “water-soluble prodrugs”, “increased solubility prodrugs” and “enhanced solubility prodrugs” in published databases including PubMed, LILACS, Scielo, Cochrane, Web of Science and Scopus.

## 2. Ester Prodrugs

The features of an ideal prodrug include the following: (a) hydrolysis resistance during absorption; (b) weak or no activity; (c) aqueous solubility; (d) good permeability through the cells; (e) chemical stability at different pHs; (f) kinetics that allow release of the parental drug [17]. Among the chemical bonds used to link the parental drug and carrier, esters have proven to be promising due to their amenability to hydrolysis both *in vivo* and *in vitro*. Some examples of the use of esters in prodrug design are discussed below.

**Figure 2:**
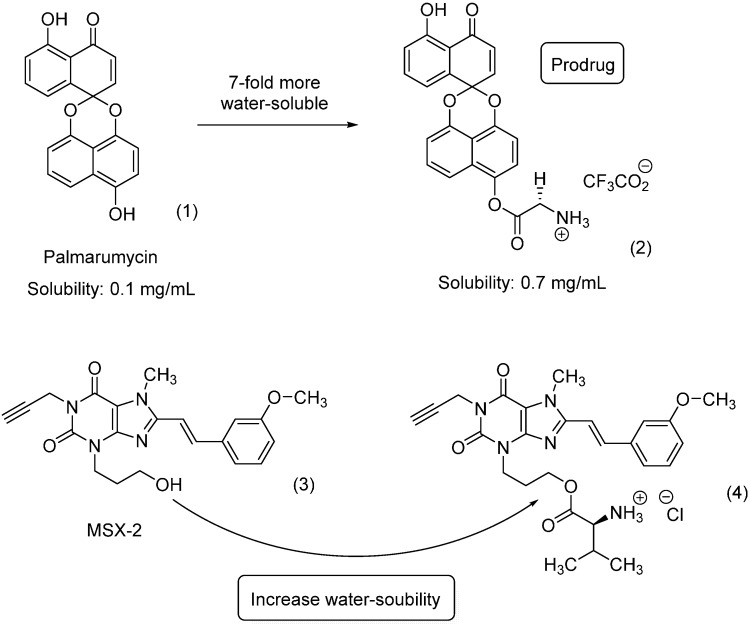
Glycyl ester and amino-acid-ester prodrugs [19,20].

The enzyme thioredoxin–thioredoxin reductase plays an important role in thioredoxin system by catalyzing the reduction of thioredoxin. Specifically, the thioredoxin system participates in protecting DNA against oxidative damage and has been implicated in several diseases, including cancer and rheumatoid arthritis [18]. Among the described inhibitors, the naphthoquinone spiroketal compound palmarumycin (**1**) has shown *in vitro* anticancer activity; however, it failed in *in vivo* assays. The authors hypothesized that the lack of activity could be due to its high lipophilicity; therefore, they designed prodrugs containing amino-esters and two morpholine analogues. The glycyl ester derivative **2** (Figure 2) was more than seven times more soluble in water than its parent drug. Despite the higher solubility compared to the parent drug, the compound did not exhibit superior activity compared to palmarumycin CP_1_ [19].

The adenosine A_2A_ receptor is a G-protein-coupled receptor expressed at high levels in blood platelets, GABAergic neurons, and cells of the thymus, the olfactory bulb and the spleen. This receptor has been widely known as a target since the approval of the drug regadenoson by the U.S. Food and Drug Administration (FDA). Among the activities described for adenosine antagonists are neuroprotective, antidepressant, and analgesic effects. In addition, this receptor has been explored as a target of new therapeutic agents to treat Parkinson’s disease [21]. Compound **3**, known as MSX-2 (Figure 2), is a potent and selective antagonist of the adenosine A_2A_ receptor [22,23]. Despite its *in vitro* activity, this molecule shows low solubility in water, hindering *in vivo* studies. Two different approaches were undertaken to resolve this issue. The first involved the introduction of a phosphate subunit to increase solubility. Phosphate prodrugs have shown high solubility in water; however, it is not readily absorbed by the oral route. The second approach involved the introduction of amino acids, specifically l-valine. The solubility in water of compound **4** (Figure 2), as evaluated by a spectrophotometric method is 7.3 mg/mL, less than that of the phosphate prodrug (9.0 mg/mL) but superior to that of the parent drug. According to the authors, the valine prodrug was stable in aqueous solution at room temperature for up to four days; however, in the presence of pig liver esterase, the compound undergoes bioconversion, releasing the parental drug, with a half-life of 6.9 min [20].

**Figure 3:**
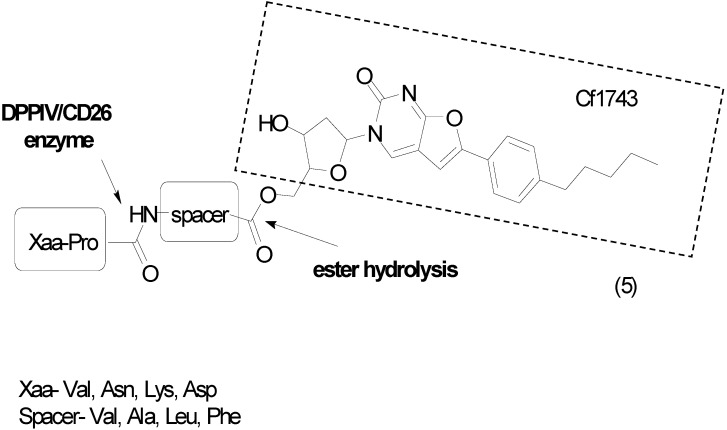
Bicyclic furanopyrimidine nucleoside analogue [25].

New water-soluble antiviral compounds also use ester amino-acid prodrugs to increase solubility with a bicyclic nucleoside subunit. This scaffold was initially described as a by-product of the condensation of 5-iodo nucleosides within terminal alkynes catalyzed by palladium; however, *in vitro* studies revealed activity against several viruses, including herpes simplex (type 1 and type 2), varicella zoster and cytomegalovirus. One of the most active compounds was the *p*-penthylphenylbicyclic nucleoside analogue Cf1743; however, its low solubility in water limits its use [24]. The prodrug approach was used to resolve this limitation, with dipeptides as carriers of Cf1743 (Val, Asn, Lys, Asp) (Figure 3). The amino-acid pro-residue is recognized by the enzyme dipeptidyl-peptidase IV (DPPIV/CD26), which is expressed in leukocytes as well as epithelial, endothelial and fibroblast cells. It was hypothesized that leukocyte enzymes would activate the prodrug. Interestingly, the authors found an analog **5** with 4000-fold greater solubility in water and 7–15-fold greater bioavailability compared to the parent drug [25].

**Figure 4:**
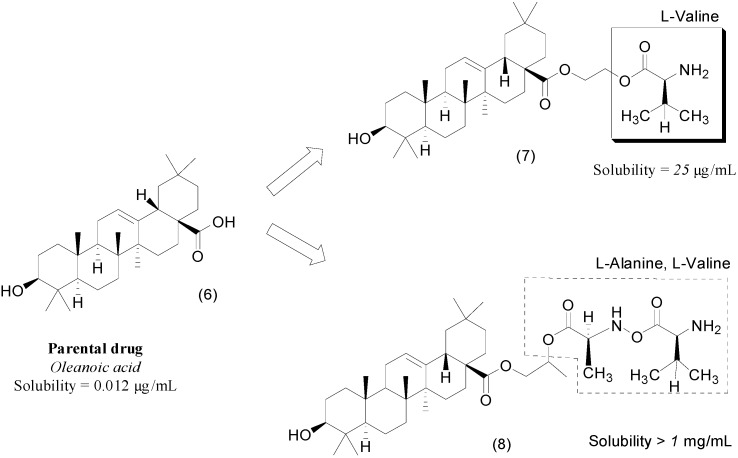
Ethylene glycol and propylene glycol prodrugs of oleanoic acid [29,30].

Amino acids were also used to improve the physicochemical properties of oleanolic acid (**6**). This triterpenoid molecule is a natural product found in Asian herbs whose pharmacological effects include anti-inflammatory, analgesic and antitumoral activities. Despite these effects, oleanolic acid has low bioavailability in rats (7%), probably due to its low solubility in water (0.0012 μg/mL). Attempts to improve the solubility using different formulations failed, probably due to its low permeability. To explore the transporters involved in the absorption of oleanolic acid, the researchers designed a series of prodrugs intended to be recognized by the PepT1 transporter. This transporter is important in drug absorption because it improves bioavailability. One commercially successful prodrug that exploits this transporter is valaciclovir, which has improved bioavailability (55%) compared to its parental drug, acyclovir (15%–20%) [26,27,28]. This transporter could also improve the absorption of oleanolic-acid derivatives. A series of novel ethylene-glycol-linked amino-acid-diester prodrugs of oleanolic acid was synthesized and tested for solubility and pharmacokinetic profile. Six analogs showed improved solubility in water. The compound **7** containing an l-valine subunit (Figure 4) was the most promising analog, exhibiting solubility greater than 25 μg/mL and increased intestinal perfusion and oral bioavailability in rats [29]. In a different paper, the same research group described a series of seven propylene glycol-linked amino-acid/dipeptide-diester prodrugs of oleanolic acid. In this study, propylene glycol (PEG) was chosen as a spacer due to its very low toxicity *in vivo*. The authors synthesized seven diester prodrugs and evaluated the influence of the linker on pharmacokinetic properties. Compound **8** was the most water soluble (up to 1.0 mg/mL; Figure 4). Compared to ethylene-glycol derivatives, the propylene-glycol series was the most soluble, stable and bioavailable and had the greatest affinity for the PepT1 enzyme [30].

**Figure 5:**
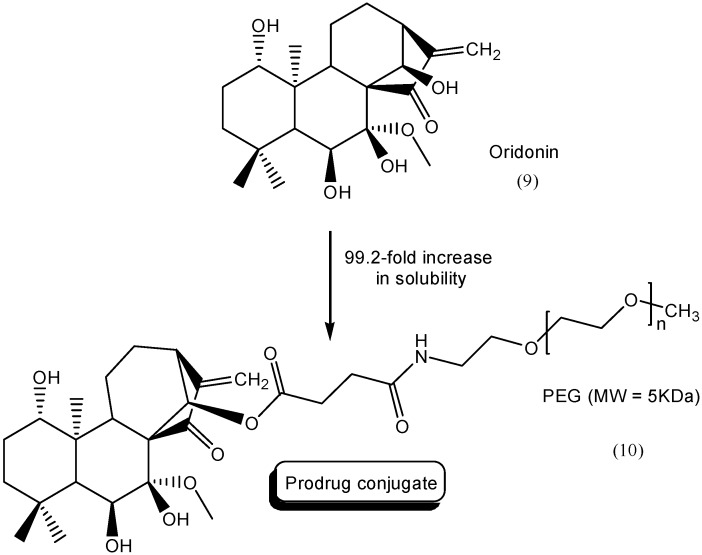
PEG prodrugs of oridonin [33].

Another terpenoid with low solubility in water whose properties were enhanced by the use of the prodrug approach is oridonin (**9**). This natural product is found in *Rabdosia rubescens*, a Chinese medicinal plant, and it exhibited antitumoral activity against several types of cancer, including leukemia [31,32]. Exploring polyethylene glycol as carrier, the authors linked oridonin to PEG, using succinic acid as a spacer. Four different molecular weights of PEGs were tested to increase solubility in water. The greatest increase in solubility was observed for a low PEG-molecular-weight (5 kDa) conjugate **10** (Figure 5). This prodrug had 99.2 times the solubility of oridonin. A chemical hydrolysis study showed a sustained-release effect for the prodrugs. *In vivo* studies using the two intermediate conjugates (10 and 20 kDa) demonstrated a successful use of this prodrug approach, as these derivatives have better pharmacokinetic profiles than oridonin [33].

**Figure 6:**
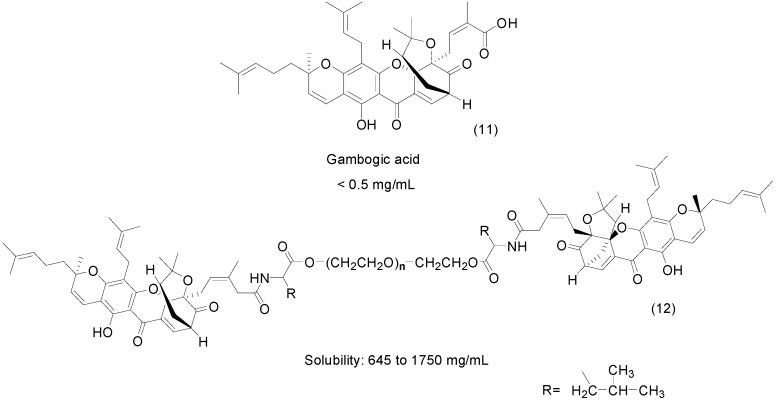
General structure of **g**ambogic-acid PEG prodrugs [35].

A similar approach exploring the use of PEG to increase solubility was carried out using another anticancer compound, gambogic acid (**11**). This natural product inhibits tumor growth and induces apoptosis in various cancer cells [34]. The authors synthesized a series of PEGylate prodrugs **12** with molecular weights of 2 kDa, 4 kDa, 10 kDa and 20 kDa using l-leucine as a spacer (Figure 6). The solubility in water ranged from 645 mg/mL to 1750 mg/mL. The most soluble prodrug contained PEG-2 kDa-l-leucine-GA. For all prodrugs, the half-life measured in plasma ranged from 1.26 to 6.12 h [35].

**Figure 7:**
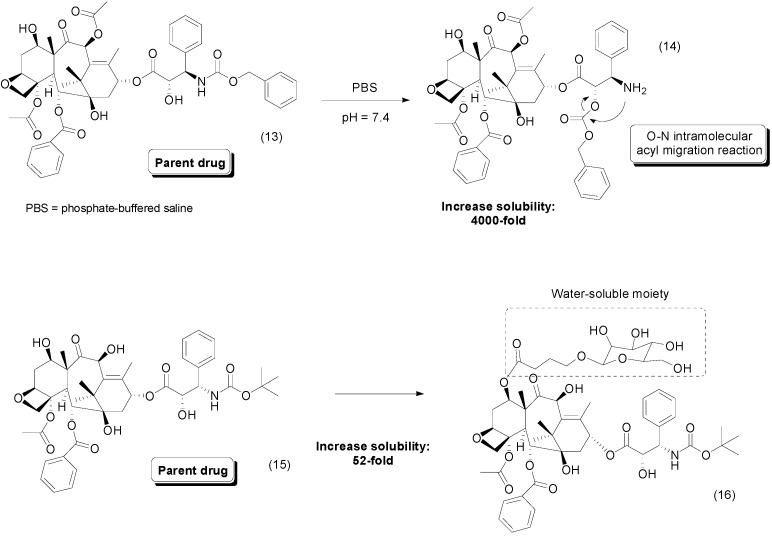
Taxoid prodrugs [37,38].

The poor solubility in water of taxoids is well established in the literature [36]. Using the prodrug approach, Skwarczynski *et al.*, synthesized prodrugs whose solubilities ranged from 0.8 to 1.1 mg/mL. Interestingly, the authors identified a novel mechanism of parental-drug release: a pH-dependent O–N intramolecular acyl migration reaction (Figure 7). This strategy increased the stability of taxoids, released the parental drug at pH 7.4 and improved drug solubility. Some of these prodrugs containing the 2′-*O*-isoform of taxoids, such as compound **14**, exhibited 4000-fold greater solubility than the parent drug **13** [37].

One approach to improve the solubility of taxoids used docetaxel-glycopyranoside ester-linked prodrugs. The compounds were prepared by chemo-enzymatic reactions using β-xylosidase, lactase and β-galactosidase. All of them demonstrated improved solubility in water compared to the parental drug, docetaxel, l and the best compound **16** showed a 52-fold increase (Figure 7). In addition, the compounds were able to release the parental drug *in vitro* and exhibited cytotoxic effects against KB and MCF-7 human cancer cell lines [38].

**Figure 8:**
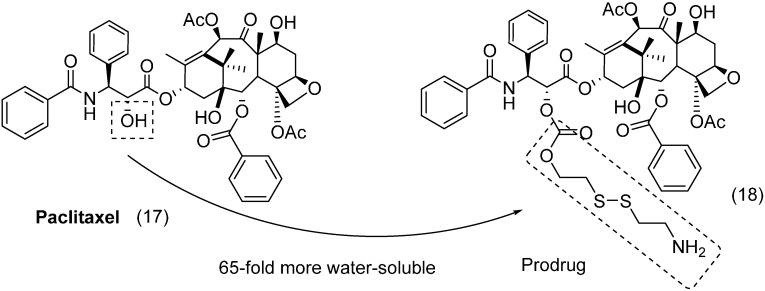
Paclitaxel-disulfide prodrug with anticancer activity [41].

A paclitaxel prodrug containing a disulfide moiety was described by Gund *et al.*, as an anticancer compound. The rationale behind the design of these molecules is based on the recognition of disulfide subunit by the enzyme glutathione, which releases the paclitaxel (**17**). High levels of this enzyme have been described in cancer cells, and one hypothesis speculates that this enzyme is involved in resistance to many anticancer drugs [39,40]. The prodrug compounds were 6–100 times more soluble than paclitaxel. Moreover, the most active compound **18** (Figure 8) was 65 times more water soluble than paclitaxel. For this molecule, superior antitumoral activity was identified in all types of cells evaluated, except for MCF10A. After oral administration, the bioavailability in mice was five-fold greater than that of the parental drug [41].

**Figure 9:**
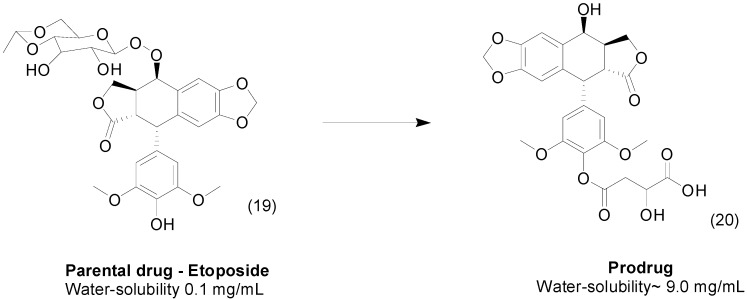
Etoposide ester prodrugs containing malic acid [42].

Etoposide (**19**) is a topoisomerase inhibitor with variable pharmacokinetics and low solubility in water. Using the prodrug approach, a series of water-soluble etoposide ester prodrugs were produced. These prodrugs with an attached malic acid demonstrated solubility in water 23 to 120-fold greater than that of the parental drug. In addition, the most active compound **20** (Figure 9), demonstrated *in vitro* cytotoxic effects comparable to those of etoposide [42].

**Figure 10:**
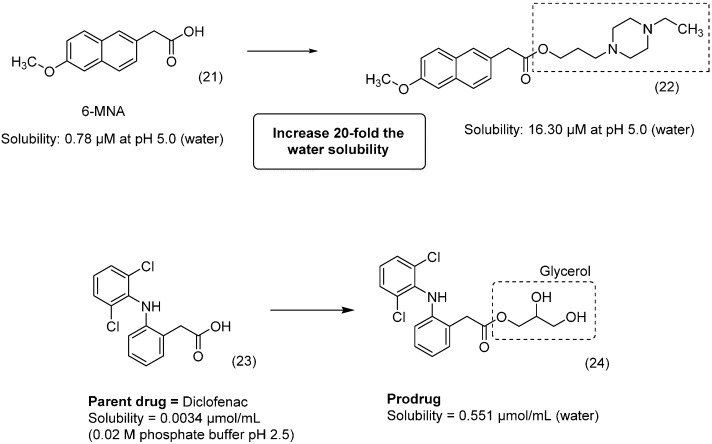
NSAID ester prodrugs [45,46].

Non-steroidal anti-inflammatory drugs (NSAIDs) are widely prescribed worldwide. The use of NSAID-ester prodrugs is extensively reported in the literature. Controversially, some authors report that the esterification of NSAIDs can reduce the adverse gastric effects by masking the carboxylic acid function; however, the changes in the pharmacokinetic profile that provide sustained release can explain in part the reduced adverse effects seen in some cases [43,44]. In many cases, the lack of solubility limits the use and worsens the adverse effects of NSAIDs. Therefore, to improve the solubility in water of the NSAID 6-methoxy-2-naphthylacetic acid (6-MNA, **21**), a series of ester prodrugs intended for percutaneous drug delivery was designed and synthesized. The authors designed piperazine derivatives attached by an ester linkage to enhance the solubility of these compounds. They identified a compound **22** that proved to be water soluble and 11.2-fold more permeable through the skin than 6-MNA at pH 7.4 (Figure 10) [45].

Exploring transdermal delivery, Lobo *et al.*, have synthesized and evaluated series of diclofenac (**23**)-ester prodrugs. The most promising compound, glycerol diclofenac ester (**24**), demonstrated better solubility, a lower partition coefficient and higher flux across the skin than its parental drug (Figure 10). *In vitro* studies using rat plasma showed rapid conversion of prodrugs to diclofenac due to the activity of esterases [46].

**Figure 11:**
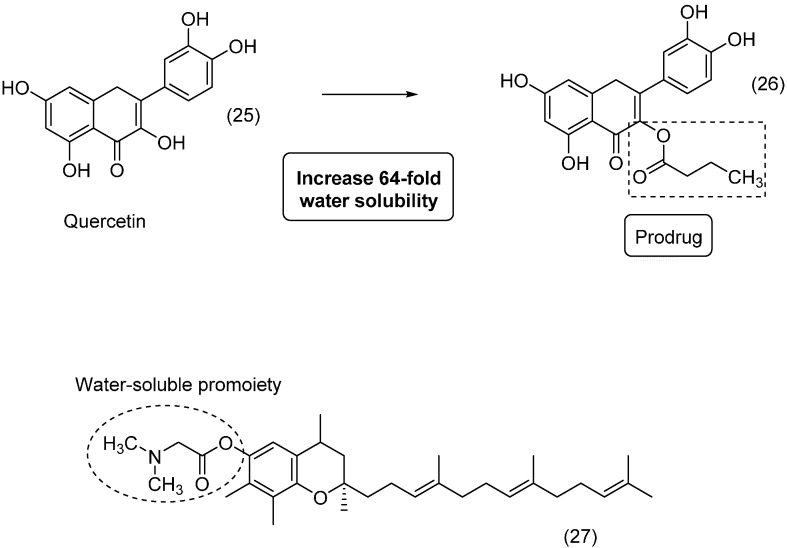
Prodrugs of quercetin and γ-T3 [48,59].

The natural product quercetin (**25**) exhibited promising anti-inflammatory properties; however, its low skin permeation limits its use [47]. Therefore, to improve the passage of quercetin through the skin, a series of prodrugs was synthesized and evaluated. The researchers observed that prodrugs with long acyl chains were less soluble than the parent drug. However, compounds with shorter acyl chains were more water soluble, with an increase of 64-fold for compound **26**. Thus, the prodrug exhibited greater solubility and skin permeation than quercetin (Figure 11) [48].

2*R*-γ-Tocotrienol (γ-T3), which is present in vitamin E, and has antioxidant activity against brain microsomes and nitric oxide and inhibits cholesterol increases and cancer proliferation [49,50,51,52,53,54,55,56,57,58]; however, γ-T3 is poorly soluble in aqueous solutions, impairing its pharmacological use. Three aminoalkylcarboxylic acid esters generated as water-soluble prodrugs of γ-T3 showed better solubility in water compared to the parental drug. Compound **27** (Figure 11) was the most promising for parenteral use, with high solubility in water, stability and rapid hydrolysis in rat and human plasma, making it a good potential alternative for cancer therapy [59].

**Figure 12:**
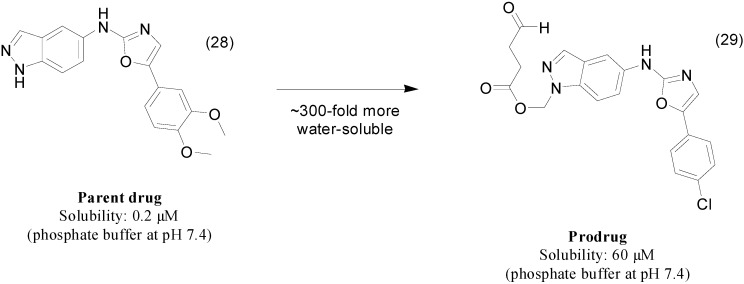
*N*-acyloxymethyl ester prodrug [60].

Screening of an in-house library was used to identify compound **28** as a promising candidate targeting Human Immunodeficiency Virus (HIV) replication; however, its poor solubility in water limits additional preclinical pharmacokinetic studies. Therefore, researchers have designed novel indazole derivatives. The most active molecule **29** (Figure 12) contains an *N*-acyloxymethyl group as a pro-moiety and was 300-fold more water soluble than its parent compound. Additionally, this prodrug was more active against HIV, exhibiting an EC_50_ of 2 μM, while the parental drug showed an EC_50_ of 3 μM [60].

## 3. Amide Prodrugs

**Figure 13:**
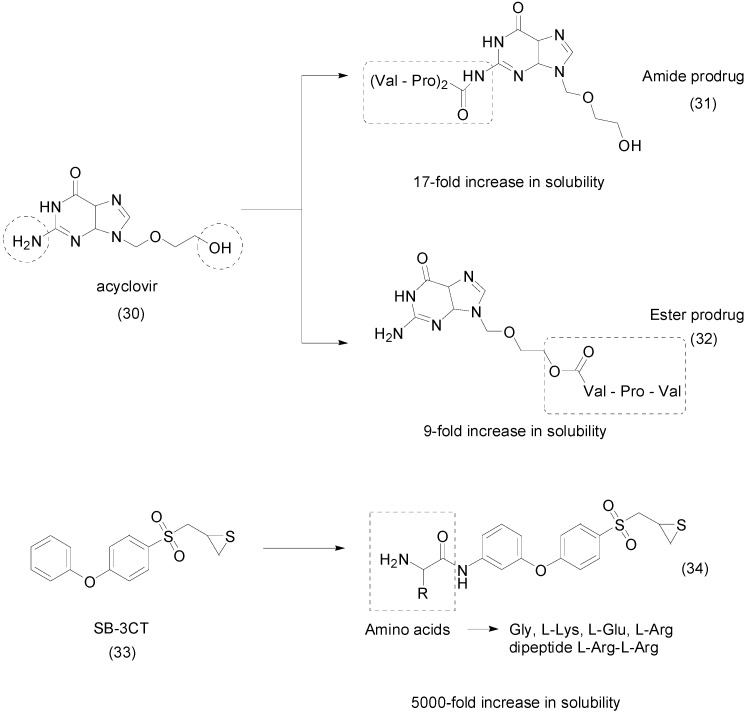
Peptide prodrugs of acyclovir and SB-3CT [62,64].

Acyclovir (**30**) is an antiviral drug that has poor solubility in water and also has low bioavailability (between 10% and 20%) [61]. To improve these characteristics, two peptide prodrugs, an amide **31** and an ester prodrug **32** were developed (Figure 13). The first was designed to release acyclovir directly through the action of enzyme dipeptidyl peptidase IV (DPPIV or CD26), and the second requires two steps to release the parental drug. Both showed a significant increase in solubility (17-fold for amide and 9-fold for ester derivatives) compared to the parent drug. Furthermore, the compounds were stable in phosphate-buffered saline (PBS) and were rapidly cleaved by plasmatic enzymes. Although the antiviral activity was not superior to that of free acyclovir, both presented good antiviral activity against herpes simplex virus type 1 and type 2 [62].

SB-3CT (**33**) is a highly selective inhibitor of the endopeptidases matrix metalloproteinases 2 and 9 (MMP-2 and MMP-9), also known as gelatinases, which have attracted interest in research due to their participation in a significant number of human pathologies such as cancer, neuronal death, atherosclerosis and aneurysms [63]. Due to the limited solubility in water of SB-3CT (2.3 μg/mL), two series of prodrugs were developed using amino acids (Gly, l-Lys, l-Glu and l-Arg) and the dipeptide l-Arg-l-Arg as pro-moieties, linked by an ester or amide group. All prodrugs showed improved solubility in water (a more than 5000-fold increase). The ester compounds were unstable in aqueous solution, human plasma and blood, while amides demonstrated resistance to fast hydrolysis. The amide prodrugs released the active drug into the blood in 30 min. The arginyl-amide prodrug **34** (Figure 13) was metabolically stable in mouse, rat and human liver microsomes. Moreover, the prodrug **34** was non-mutagenic in an Ames assay [64].

**Figure 14:**
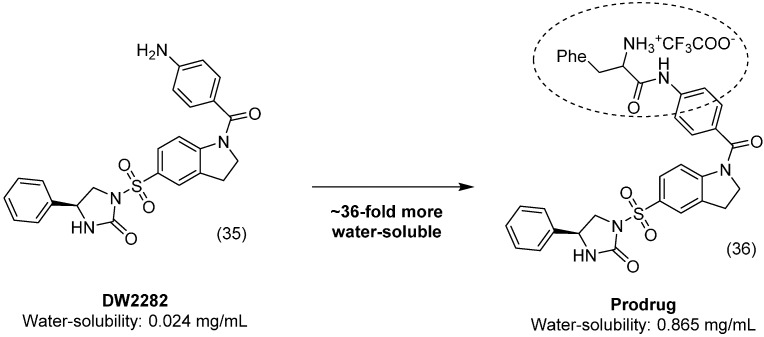
Prodrug derivative of DW2282 [66].

DW2282 (**35**) is a potent antiproliferative compound with a broad spectrum of action against various cancer cell lines, but in preclinical assays, this analog showed gastrointestinal toxicity [65]. A series of novel amino-acid conjugates were designed to improve the aqueous solubility of these analogs and prevent toxic effects. All synthesized compounds were more soluble and three were more active than the parental drug. The most promising analog **36** (Figure 14) had a good reconversion rate in human plasma and good bioavailability by the oral route in mice. Moreover, this molecule was more active and approximately 36-fold more soluble than DW2282 [66].

Bone tissues are difficult to treat because of limited drug delivery and retention and low sanguineous flow [67]. Hydroxyapatite (HA), a constituent of bones, can be a target for these drugs through the interaction between negative groups and the calcium ions in HA [68,69,70,71]. Researchers designed and evaluated the solubility and pharmacokinetic profile of novel dendritic naproxen-peptide prodrugs, specifically l-aspartate and l-glutamate.

**Figure 15:**
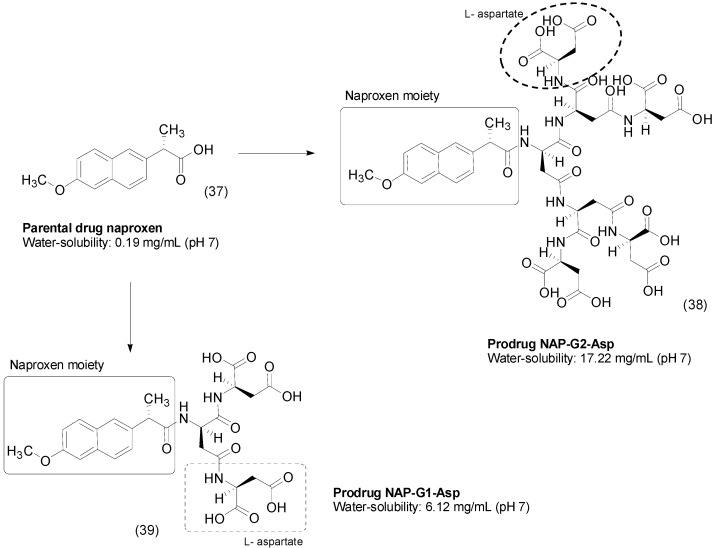
Dendritic naproxen-peptides prodrugs [72].

Their solubility in water was evaluated at three different pH values, and the prodrugs were more soluble at increased pH. The authors proposed that the solubility of the analogs can be related to surface area and carboxyl groups in dendritic peptides; in this way, the second-generation molecules have more interactions with water and thus are more water soluble than the first-generation drugs **39** (Figure 15). The most promising analog NAP-G2-Asp (**38**) attached to l-aspartate subunits showed an approximately 90-fold increased solubility in water at pH 7. In the HA-binding assay, NAP-G2-Asp showed 80% binding activity in 25 mg/mL in PBS. In 50% human plasma it acted as a prodrug, rapidly and effectively releasing the parent drug [72].

**Figure 16:**
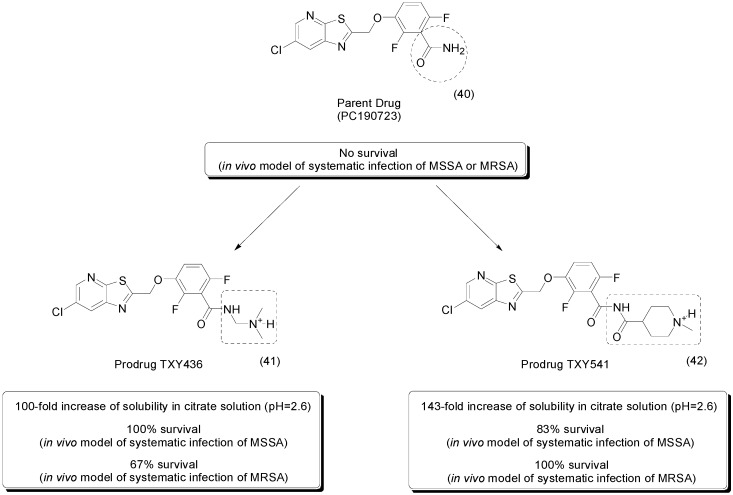
Benzamide and carboxamide prodrugs of PC190723 [73,74].

A benzamide derivative, PC190723 (**40**, Figure 16), was proposed as a potent antimicrobial agent targeting the FtsZ protein. This enzyme plays a key role in bacterial cell division, and studies suggest that it is a promising new target for antibiotic development. To overcome its poor formulation, two new prodrugs were developed. The first, the compound TXY436 (**41**, Figure 16) is a *N*-Mannich base derivative that was 2.8-fold more soluble than the parent drug in PBS (pH = 7.4) and 100-fold more soluble in 10 mM citrate solution (pH = 2.6). The latter is suitable for *in vivo* drug administration. Moreover, this compound is stable in citrate solution and is rapidly converted to the parent drug under physiological conditions (pH = 7.4). In addition, it maintained anti-staphylococcal activity due to *in vitro* FtsZ inhibition and showed 73% oral bioavailability. *In vivo* studies demonstrated that the prodrug was effective in a murine model of peritonitis with systematic infection with methicillin-susceptible *Staphylococcus aureus* (MSSA, survival of 100%) and methicillin-resistant *Staphylococcus aureus* (MRSA, survival of 67%), while the parent drug was not effective in this model [73].

The second prodrug TXY541 (**42**, Figure 16) is a carboxamide derivative with increased solubility in water (143-fold in citrate solution). Interestingly, the solubility in PBS decreased, to almost half the solubility of the parental compound. The stability study revealed that TXY541 is stable in citrate solution and is converted under physiological conditions to PC190723, but at slower rates than the parental compound is **41**. In mouse serum, the conversion occurred faster, which suggest an enzymatic conversion. The oral bioavailability of the prodrug **42** was 29.6%, and *in vivo* evaluation showed that this analog was effective against systematic infection with MSSA (83% survival) and MRSA (100% survival) [74].

## 4. Carbamate Prodrugs

**Figure 17:**
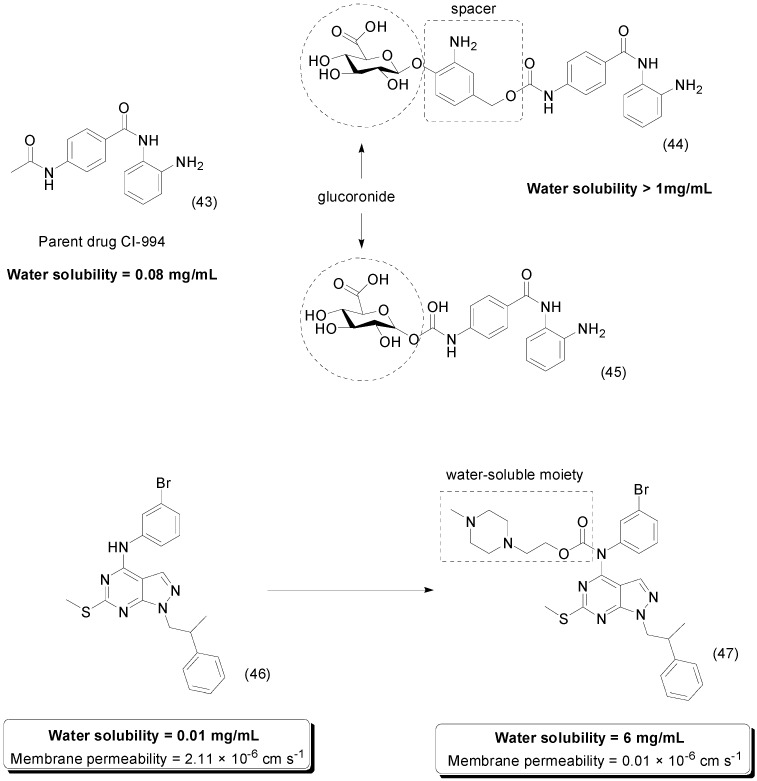
Glucuronide and pyrazolo[3,4-*d*]pyrimides prodrugs [77,78].

The benzamide compound CI-994 (**43**) is a potent inhibitor of histone deacetylases (HDACs). HDACs have an essential role in the regulation of gene expression, and several types of inhibitors exhibit antitumor activity [75,76], although adverse effects and poor solubility in water limit its use. Two glucuronide prodrugs, one (compound **44**) linked to the glucuronide moiety by a spacer and the other linked directly by a carbamate group (compound **45**), were used to resolve these limitations (Figure 17). While the solubility of CI-994 is 0.08 mg/mL, those of the prodrugs were greater than 1 mg/mL. Both compounds were stable at different pH values (2.1 and 7.0). In addition, the prodrugs were evaluated *in vitro* using an antiproliferative assay with NCI-H661 non-small cell lung cancer cells. The results demonstrated that this compound, when incubated with β-glucuronidase, showed the same antiproliferative activity (IC_50_ = 20 μM) shown by CI-994. Without the enzyme, the prodrugs had decreased cytotoxicity, which may decrease the toxic effects of the parent drug [77].

Pyrazolo[3,4-*d*]pyrimides compounds are able to inhibit oncogenic tyrosine kinases and may be used to treat cancer. In general, pyrazolo[3,4-*d*]pyrimides compounds have low solubility in water [79]. Prodrugs of pyrazolo[3,4-*d*]pyrimidine compounds were designed to enhance its pharmacokinetic properties using a water-soluble *N*-methylpiperazino promoiety linked by a *O*-alkyl carbamate linker. The prodrug **47** showed 600-fold improvement in solubility compared to the parent drug **46** (Figure 17). *In vitro* assay showed good increase in passive membrane permeability, from 0.01 × 10^−6^ cm·s^−1^ to 2.11 × 10^−6^ cm·s^−1^. In addition, this compound was more cytotoxic compared to the parental drug; however, the mechanism of action is still not well characterized [78].

Photodynamic therapy is a non-invasive treatment for several types of cancer and infectious diseases [80]. This strategy consists in administration of a sensitizer and non-mutagenic substance that will be irradiated only in the affected area, where it will be activated by a specific wavelength of light [81]. This tool can be considered a prodrug approach, in that it uses the light to convert the photosensitizer (prodrug) to an active compound and in that both the selectivity and the solubility of the photosensitizer are important in design [80].

**Figure 18:**
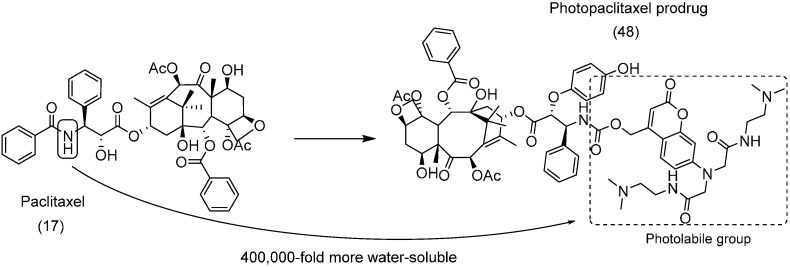
Photopaclitaxel prodrug [82].

Three photopaclitaxel prodrugs were generated by linking paclitaxel (**17**) with a coumarinic derivative. The most soluble compound **48** showed at least 400,000-fold higher solubility in water compared to paclitaxel (Figure 18). This same compound was stable under physiological and storage conditions. In a photoconversion assay, the carbamate prodrug **48** rapidly generated paclitaxel with minimal tissue damage under 365 nm UV-A light irradiation at low power [82].

## 5. Carbonate Prodrugs

**Figure 19:**
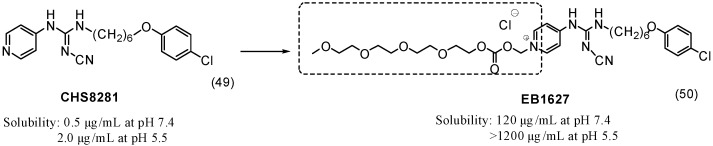
CHS8281 prodrug [83].

In an attempt to improve the solubility of an anticancer compound, CHS8281 (**49**), a series of prodrugs was synthesized using a carbonate group to link a tetraethylene-glycol moiety to the parental drug. The prodrug EB1627 (**50**) was 600-fold more soluble than the parental drug at pH 5.5 (Figure 19). The selection of the carbonate group provided the required hydrolytic labiality and rapid release of the active drug *in vivo* [83].

**Figure 20:**
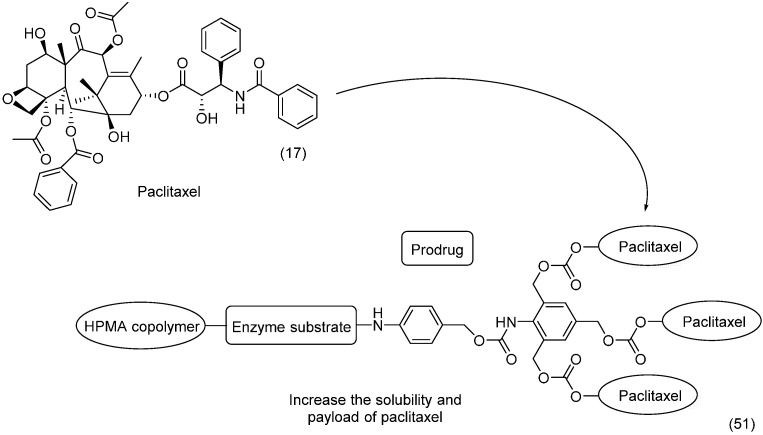
Copolymer-paclitaxel conjugate [84].

To overcome the poor solubility of paclitaxel (**17**) and release the drug selectively into malignant tissue, an *N*-(2-hydroxypropyl)-methacrylamide (HPMA) copolymer-drug conjugate **51** with an AB_3_ self-immolative dendritic linker was designed. The HPMA was used as a water-soluble group, and the dendritic peptide linked the HPMA to the parent drug (Figure 20). This enzyme-cleavable linker served as a platform with a triple payload of paclitaxel attached by a carbonate group [84]. Interestingly, HPMA not only showed improved solubility but also selectively accumulated in tumors due to its enhanced permeability and retention [85].

## 6. Ether Prodrugs

**Figure 21:**
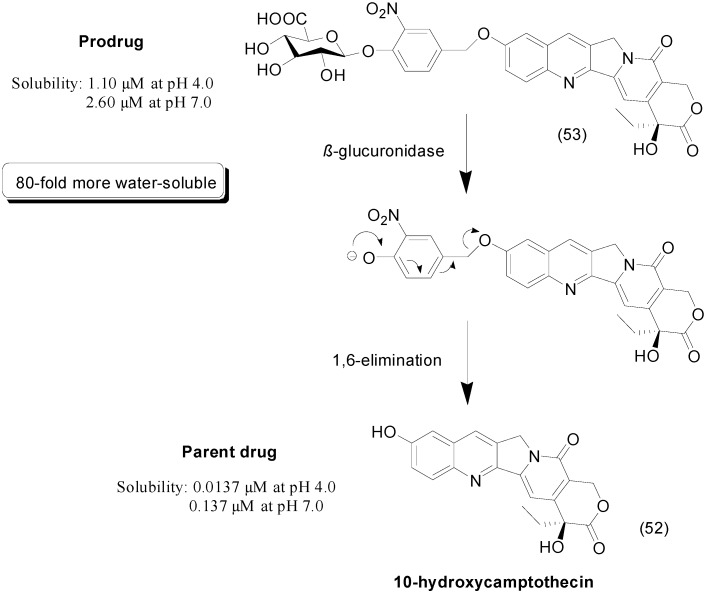
Prodrug release based on an enzymatic cleavage followed by a 1,6-elimination reaction [88].

A glucuronide prodrug of the anticancer compound 10-hydroxycamptothecin (**52**) was reported by Leu *et al.* The authors used the prodrug approach to improve the solubility of the parental drug, which showed anticancer activity against various cancer cell lines [86,87]. To improve the solubility of 10-hydroxycamptothecin, a prodrug was designed using glucuronic acid as a water-soluble moiety linked by a 3-nitrobenzyl spacer. The compound **53** was 80-fold more soluble than the parental drug. The prodrug was activated by enzymatic cleavage followed by a 1,6-elimination reaction to release the parental drug (Figure 21) [88].

**Figure 22:**
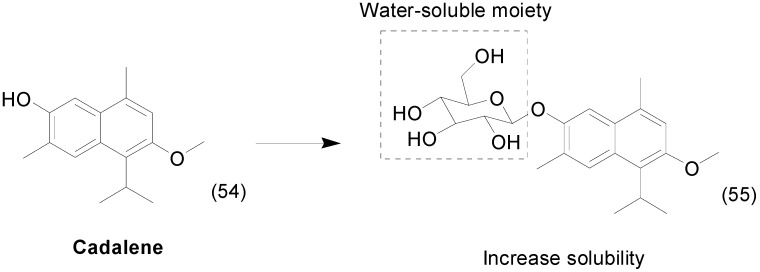
Cadalene prodrug with improved solubility in water [90].

Cadalene (**54**) is a flavonoid isolated from *Zelkova serrata Makino*. This molecule has a wide range of biological activities, including anticancer activities; however, its low solubility in water is a drawback [89]. Glycosylated cadalene derivatives linked by ether orethoxy spacers were synthesized as prodrugs. Prodrug **55** (Figure 22) exhibited the best solubility profile. *In vivo*, treatment with cadalene did not affect tumor volume, but treatment with the glycosylated prodrug reduced tumor volume by 50% compared to the control group. The authors hypothesized that the better activity of the prodrug could be due to its better solubility compared to cadalene [90].

## 7. Imine Prodrugs

**Figure 23:**
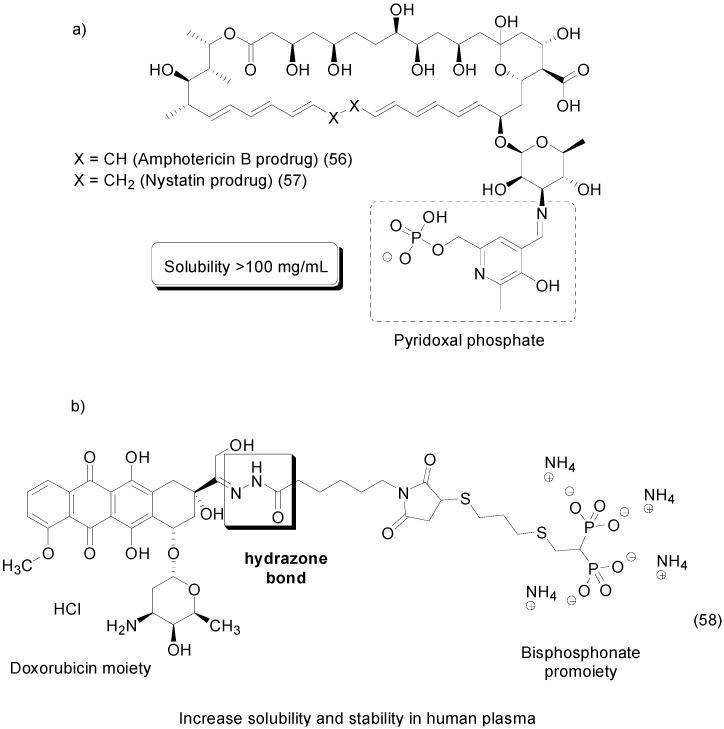
(**a**) Amphotericin B and nystatin prodrugs; (**b**) Bisphosphonate doxorubicin prodrug [91,92].

Amphotericin B and nystatin prodrugs containing the pyridoxal phosphate as a water-soluble moiety were synthesized and evaluated. An imine group was used to allow the attachment of both subunits, and high water-soluble prodrugs **56** and **57** with solubilities up to 100 mg/mL were characterized (Figure 23) [91].

Another paper described the synthesis of a novel bisphosphonate doxorubicin prodrug to treat bone metastases. To improve the solubility of doxorubicin, the water-soluble group bisphosphonate was attached to the parental drug by a hydrazone acid-sensitive bond. Moreover, the bisphosphonate moiety acts as a bone-targeting ligand, improving the drug’s selectivity. The prodrug **58** showed considerably higher stability at pH 7.4, with approximately 12% of the doxorubicin being released after 18 h of incubation (Figure 23) [92].

**Figure 24:**
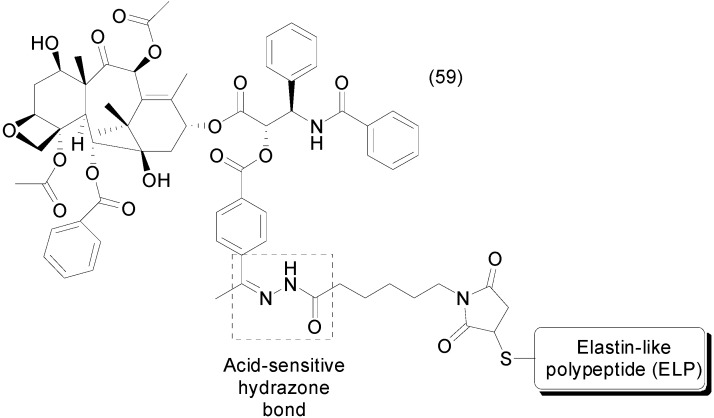
Paclitaxel-biopolymer-conjugated prodrug [93].

Synthesis of a soluble paclitaxel prodrug using an acid-sensitive hydrazone bond was reported by Moktan *et al.* The authors used a biopolymer elastin-like polypeptide (ELP) as a water-soluble group to improve the solubility of paclitaxel. ELP also enables thermal targeted delivery of paclitaxel, as hyperthermia above a specific transition temperature at the site of a tumor causes ELP to aggregate and accumulate, thus increasing the local concentration of the drug. The ELP paclitaxel prodrug **59** was able to increase solubility and cytotoxicity against a paclitaxel-resistant breast cancer cell line (Figure 24) [93].

## 8. Phosphate Prodrugs

Phosphate ester compounds are relatively stable when free in metabolic surroundings because they have a low p*K*a, between 1 and 2. At a physiological pH of 7.0–7.4, the compounds are permanently deprotonated and therefore negatively charged but are readily activated upon complexation to various counterions in an enzyme’s active site [94,95]. Intestinal alkaline phosphatases play an important role in drug metabolism by cleaving phosphate prodrugs and releasing the parental drugs [96]. Within the cell, the regeneration of the parental drug from the phosphate prodrug is difficult to determine. In most cases, this process is assumed to involve a specific enzyme or enzymes such as an esterase, an amidase, a phosphatase, or even a redox process [95,97,98]. Therefore, the phosphate-prodrug approach has been successfully used to enhance the solubility and bioavailability of the parental drug.

**Figure 25:**
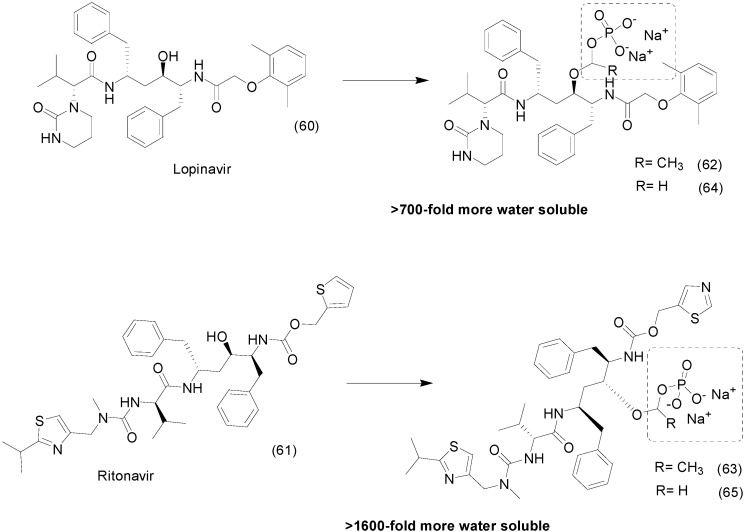
Structures of the prodrugs of lopinavir and ritonavir [99].

Degoey *et al.*, synthesized a series of prodrugs of lopinavir (LPV, **60**) and ritonavir (RTV, **61**), HIV protease inhibitors. The oxymethylphosphate and oxyethylphosphate prodrugs (**62/63** and **64/65**, respectively) had more than 700 and 1600-fold enhanced solubility in water compared to LPV and RTV, respectively (Figure 25). Pharmacokinetic studies in rats and dogs showed high plasma levels of the parent drugs after administration via the oral route [99].

**Figure 26:**
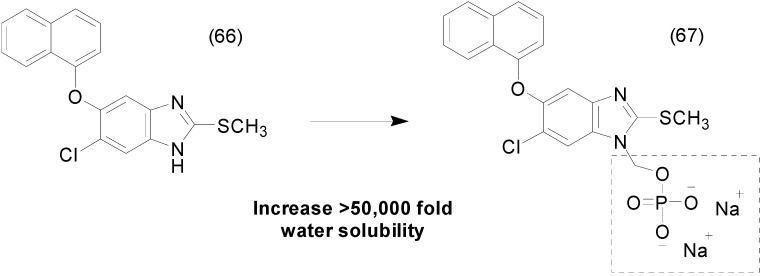
Benzimidazole phosphate prodrug [100].

Another example of water soluble prodrug with phosphate group was synthesized by Flores-ramos *et al.*, using as prototype the compound α-6-chloro-2-(methylthio)-5-(naphthalen-1-yloxy)-1*H*-benzo[*d*]imidazole (**66**), a benzimidazole derivative. The solubility of the prodrug **67** was increased 50,000-fold when compared to its precursor by linking a disodium phosphate to the structure (Figure 26). *In vitro* and *in vivo* assays demonstrated that the prodrug have faciolicidal activity; however, administered dose *in vivo* was lower than the parent drug [100].

**Figure 27:**
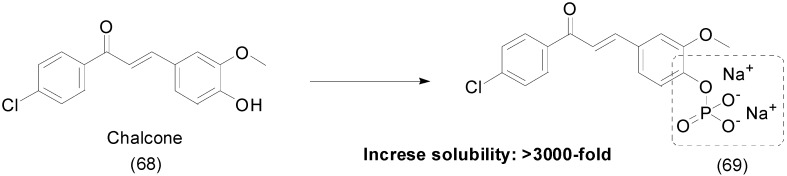
Chalcone-phosphate prodrugs [102].

The chalcone family is known to prevent CXCL12 chemokine from binding to its CXCR4 or CXCR7 receptors, preventing inflammatory reactions, but their poor solubility in water limits their use (9 μM/mL) [101]. To overcome this problem, chalcone prodrugs **68** were synthesized with different functional groups, such as phosphate, an l-seryl, and a sulfate. The phosphate prodrug **69** (Figure 27) showed a greater increase in solubility, to at least 3000 times greater solubility than the parent chalcone. These results allowed low-dose intranasal administration, with ≥50% inhibited eosinophil recruitment in the airways at a dose as low as 30 nmol/kg without any signs of toxicity [102].

**Figure 28:**
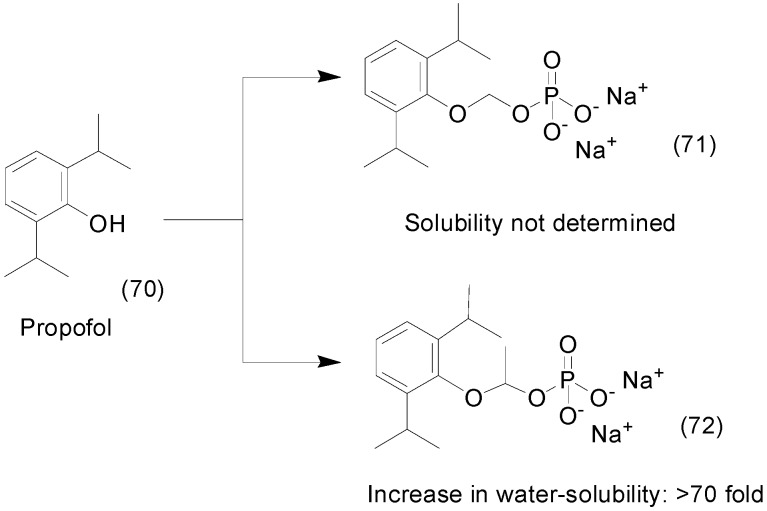
Propofol prodrug [103].

Propofol (**70**) is another drug with high lipophilicity and is administered in an oil-in-water emulsion. To overcome this drawback, phosphate prodrugs of propofol have been generated using a phosphate group attached directly to the hydroxyl group of propofol with oxymethyl (compound **71**) and ethylenedioxy moieties (compound **72**) as spacers linked to a phosphate group (Figure 28). All these prodrugs demonstrated stability and enhanced solubility in water. Solubility of the ethyl dioxy phosphate derivative **72** was increased more than 70-fold compared to propofol. Furthermore, neither prodrug produced formaldehyde, a toxic subproduct, and thus both represent useful water-soluble prodrugs suitable for i.v. administration [103].

**Figure 29:**
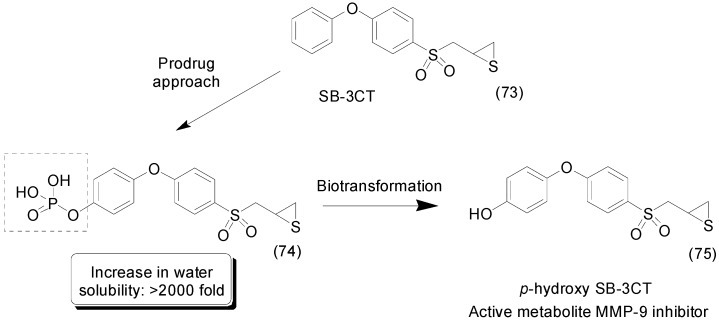
SB-3CT prodrug and its active metabolite [104].

SB-3CT (**73**) is a selective inhibitor of matrix metalloproteinases 2 and 9 that is active in the treatment of traumatic brain injury (TBI) [63]. Its phosphate prodrug **74** (Figure 29) showed more than 2000-fold enhanced solubility in water. The prodrug was readily hydrolyzed to the active *p*-hydroxy SB-3CT (**75**), which crossed the blood-brain barrier and reached a therapeutic concentration in the brain. In an *in vivo* assay in TBI mice, the prodrug significantly decreased brain-lesion volume and improved neurological outcomes [104].

**Figure 30:**
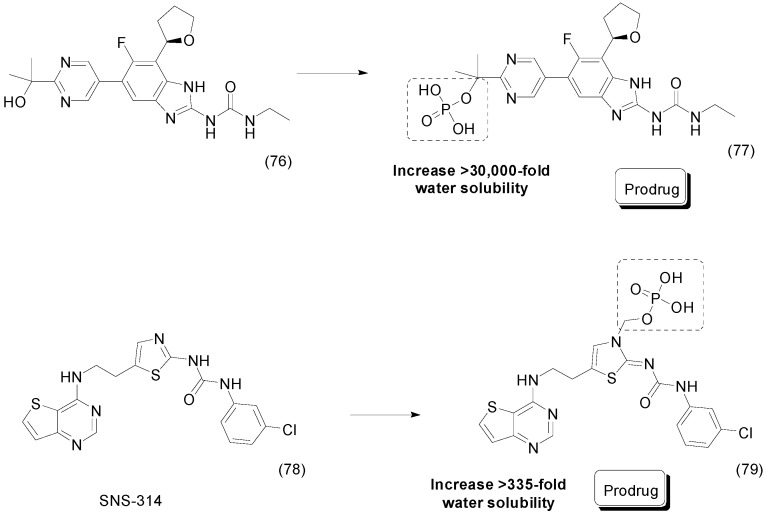
Benzimidazole and SNS-314 phosphate prodrugs [107,108].

Compound **76** is a benzimidazole derivative with antibacterial activity that inhibits DNA gyrase and topoisomerase IV [105,106]. Due to its poor solubility in water, Dowd *et al.*, synthesized a benzimidazole phosphate-ester prodrug. The compound **77** was up to 30,000-fold more water soluble than the parent drug at physiological pH (Figure 30). Interestingly, the phosphate-ester prodrug was much less potent *in vitro* against all bacteria strains tested. Nevertheless, in the target-level assay, the prodrug exhibited similar activity to the parent drug against *S. aureus* gyrase and topoisomerase IV [107].

Aurora serine/threonine kinases have been described as a promising anti-cancer target [109]. To overcome the poor solubility in water of SNS-314 (**78**), an aurora kinase inhibitor, a prodrug was designed with several moieties attached to the parent drug, such as acyl-oxymethylene, amine-containing acyl-oxymethylene and phosphate group. Prodrug **79**, a phosphate-ester derivative, was 335-fold more water soluble than the parent drug (Figure 30). Moreover, although the acyl-oxymethylene derivatives showed increased solubility (8–20-fold), they were much less effective than the phosphate-ester prodrug [108].

## 9. Other Chemical Prodrugs

**Figure 31:**
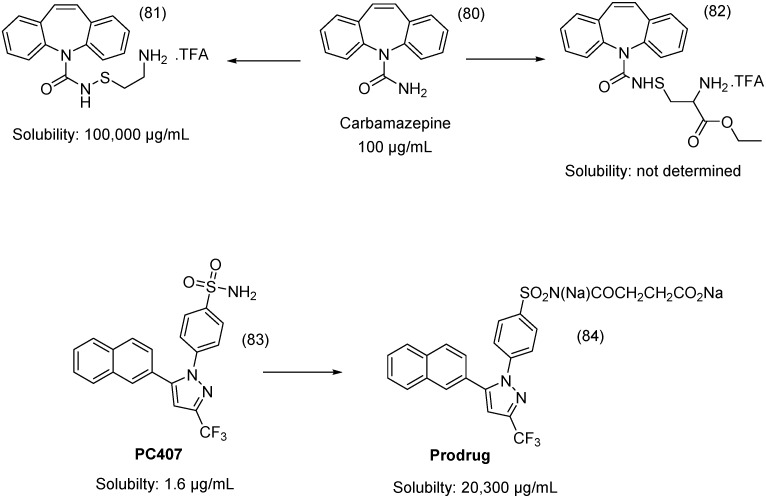
Carbamazepine and PC407 prodrugs [111,112].

Carbamazepine (CBZ) (**80**) is an effective anticonvulsant drug that causes less sedation and cognitive impairment than other anticonvulsants; however, its aqueous solubility is 120 μg/mL, hindering intravenous administration [110]. To overcome this problem, CBZ prodrugs **81** and **82** with improved solubility were synthesized (Figure 31). The aqueous chemical stability of **81** was superior to that of **82**. Compound **81** was dissolved in water, with apparent solubility in excess of 100 mg/mL (final pH value of 2.6). An *in vivo* pharmacokinetic assay with i.v. administration demonstrated that compound **81** was rapidly converted to CBZ [111].

The sulfonamide group was also utilized to develop a cyclooxygenase 2 (COX-2)-inhibitor prodrug of PC407 (**83**) for parenteral administration (Figure 31). The prodrug showed highly improved solubility, from 1.6 μg/mL to 20.3 mg/mL. Prodrug **84** showed analgesic activity *in vivo* and aqueous stability and is a promising candidate COX-2 inhibitor for injectable formulation [112].

**Figure 32:**
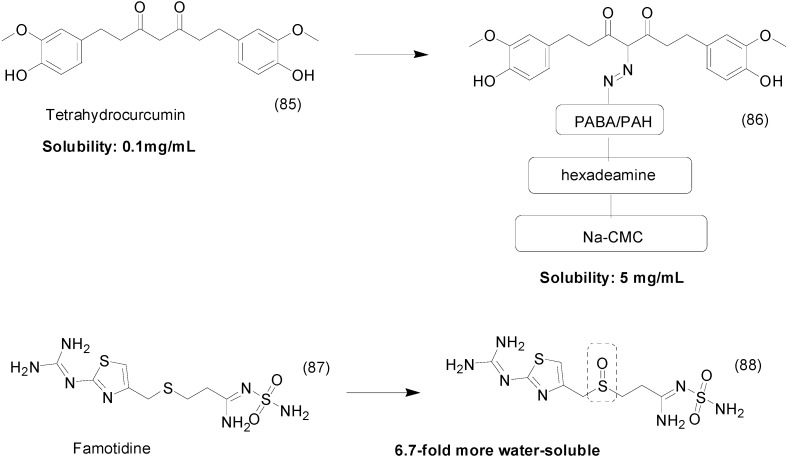
Tetrahydrocurcumin and famotidine prodrugs [116,118].

Tetrahydrocurcumin (THCur) (**85**) is a metabolite of curcumin that shows antioxidant and anticancer activities [113,114,115]. A series of THCur prodrugs was synthesized using carboxymethylcellulose (CMC) as a water-soluble group linked by an azo bond. 4-Amino-THCur (**86**, Figure 32) has shown increased solubility in water up to 5 mg/mL and was released in the colon via azoreductase enzymes of colonic bacteria (up to 62% within 24 h). In human colon adenocarcinoma cell lines (HT-29), the prodrug was cytotoxic, with a IC_50_ of 28.67 ± 1.01 μg/mL, and selective to these cells, unlike curcumin, which was non-selective [116].

Famotidine (**87**) is a histamine H2-receptor blocker used to treat and prevent ulcers [117]. Vijayaraj *et al.*, synthesized a water-soluble prodrug of famotidine by introducing a sulphoxide group. This modification (compound **88**) increased solubility in water 6.7-fold compared to famotidine (Figure 32). The prodrug was stable at pH 1.2 and 7.4 and might be effective in ulcer therapy [118].

## 10. Marketed Prodrugs

From 2005 to 2015, several prodrugs were discovered and launched in the market (Table 1). Some of these drugs are summarized in Table 1 and will be discussed below.

**Table 1:** Prodrugs launched in the USA, 2005–2015 ^a^.

Year	Prodrug Name	Chemical Structure	Indication
2005	Nelarabine Arranon^®^ GlaxoSmithKline plc	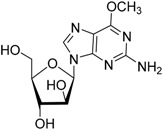	Lymphoblastic leukemia
2006	-	-	-
2007	Lisdexamfetamine dimesylate Vyvanse^®^ New River, Inc.	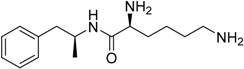	Attention–deficit/hyperactivity disorder
	Temsirolimus Torisel^®^ Wyeth Pharm., Inc.	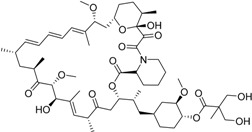	Advanced renal cell carcinoma
2008	Fesoterodinefumarate Toviaz^®^ Pfizer, Inc.	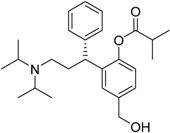	Overactive bladder disorder
	Fospropofoldisodium Lusedra^®^ ElanPharm. plc	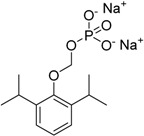	Monitored anesthesia care sedation. # discontinued
2009	Prasugrel Effient^®^ Eli Lilly (developed with Daiichi Sankyo)	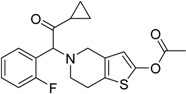	Prevention of thrombotic cardiovascular complications in acute coronary syndromes
	Romidepsin Istodax^®^ Gloucester Pharm.	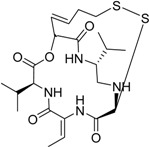	Cutaneous T cell lymphoma
2010	Dabigatranetexilate mesylate Pradaxa^®^ Boehringer Ingelheim GmbH	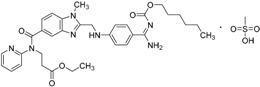	Thromboembolism acute coronary syndrome
	Fingolimod Gilenya^®^ Novartis International AG	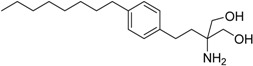	Multiple sclerosis [sphingosin-1-phosphate (S1P) agonist with cannabinoid antagonist]
	Ceftarolinefosamil Teflaro^®^ Forest Laboratories, Inc.	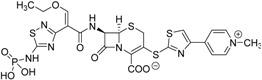	Bacterial skin infection
2011	Abiraterone acetate Zytiga^®^ Janssen Biotech, Inc.	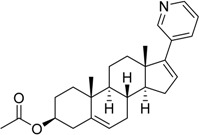	Hormone-refractory prostate cancer (17-α-hydrolase/C17,20lyase)
	Azilsartanmedoxomil Edarbi^®^ Takeda Pharm.	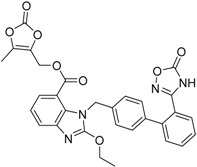	Hypertension (angiotensin II antagonist)
	Gabapentin encarbil Horizant^®^ GlaxoSmithKline plc	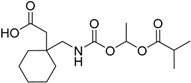	Restless leg syndrome (GABA and Ca channel modulator) new indication
2012	Tafluprost Zioptan^®^ Merck & Co., Inc.	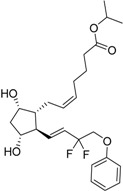	Elevated intraocular pressure (prostaglandin analog)
2013	Dimethyl fumarate Tecfidera^®^ Biogen Idec, Inc.	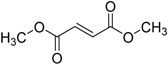	Relapsing multiple sclerosis
	Eslicarbazepineacetate Aptiom^®^ Bial-Portela & Ca. S.A.	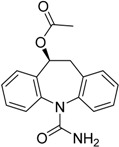	Anticonvulsant (partial-onset seizures)
	Sofosbuvir Sovaldi^®^ Gilead Sciences, Inc.	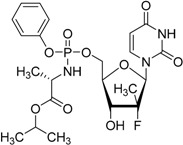	Treatment of hepatitis C virus (HCV) infection
2014	Droxidopa Northera^®^ Lundbeck A/S	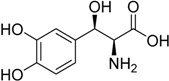	Neurogenic orthostatic hypotension
	Tedizolidphosphate Sivextro^®^ Cubist Pharma., Inc.	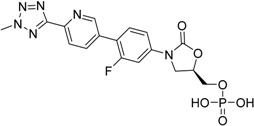	Acute bacterial skin and skin structure infections
2015	Isavuconazonium sulfate Cresemba^®^ Astellas Pharma, Inc.	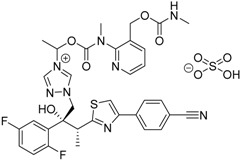	Invasive aspergillosis and invasive mucormycosis

^a^ The table does not include the launch of reformulations or new indications of already-marketed prodrugs. Drugs approved till the first half 2015.

Eslicarbazepine (ESL) is an antiepileptic prodrug developed from oxcarbazepine and approved by the European Medicines Agency (EMA), the Food and Drug Administration and Health Canada as an adjunctive therapy in adults with partial-onset seizures. The development of the ESL acetate prodrug was based on the observation that the active *S*-enantiomer of ESL is produced after biotransformation at a rate 20-fold higher than the inactive *R*-enantiomer [119,120].

Fospropofol disodium, a phosphate ester prodrug of propofol, was approved by the FDA in 2008 as a sedative–hypnotic agent for monitored anesthesia-care sedation in patients who are undergoing diagnostic or therapeutic procedures. The aqueous solubility of fospropofol provides an advantage over propofol, which is available only as a lipid-containing oil-water emulsion. These advantages include lack of pain on injection, different pharmacokinetic profile due to *in vivo* conversion of fospropofol to propofol and rapid recovery from sedation [121,122].

Ceftaroline fosamil, a phosphoramidate prodrug launched in 2011 for the treatment of bacterial skin infections and bacterial pneumonia, showed 50-fold enhancement of solubility relative to the parental drug, thus enabling formulation of a marketable injectable solution [123].

Tedizolid phosphate, which has been marketed for the treatment of acute bacterial skin and skin-structure infections since 2014, is a phosphate-ester prodrug of the active compound tedizolid. The additional phosphate group provides improved aqueous solubility and thus bioavailability [124,125].

## 11. Conclusions

The prodrug approach has been a successful tool for improving solubility in water, as can be observed from several publications that showed up to 400,000-fold increased solubility compared to the parent drug, as described herein. This approach can make it possible to avoid discarding promising active prototypes or drugs with therapeutic uses limited by poor solubility. The rational selection of the adequate pro-moiety and the type of linkage, (e.g., ester, amide, carbamate and phosphate), may determine the prodrug selectivity, toxicity, and ideal bioconversion profile.

Furthermore, the prodrug approach could be viewed as an alternative in the early phases of drug discovery. This technique may be used to modulate pharmacokinetic properties (absorption, distribution, metabolism and excretion), as well as poor water solubility, a critical step in pre-clinical phases. The majority of prodrugs presented herein were esters and amides because esterases and amidases could activate them, releasing the parent drug. Amino acids were the most-used water-soluble pro-moieties and, as demonstrated by several reviewed authors, efficiently increase solubility in water. Nevertheless, several other chemical groups are represented herein to a lesser degree, such as glycol groups (e.g., polyethylene glycol and ethylene glycol) and glycosides.

Several prodrugs were approved in the last 10 years by the FDA, although not all prodrugs were designed to increase solubility. Nevertheless, many prodrugs were designed for this purpose, such as tedizolid phosphate, ceftaroline fosamil and fospropofol disodium. Therefore, the prodrug approach is an important tool in rational drug design to improve drug solubility in water.
